# A splicing site change between exon 5 and 6 of the nuclear-encoded chloroplast-localized *HvYGL8* gene results in reduced chlorophyll content and plant height in barley

**DOI:** 10.3389/fpls.2023.1327246

**Published:** 2023-12-12

**Authors:** Xue Xia, Lei Liu, Kangfeng Cai, Xiujuan Song, Wenhao Yue, Junmei Wang

**Affiliations:** ^1^ Key Laboratory of Digital Dry Land Crops of Zhejiang Province, Zhejiang Academy of Agricultural Sciences, Hangzhou, China; ^2^ Zhejiang Academy of Agricultural Sciences, National Barley Improvement Center, Hangzhou, China; ^3^ College of Advanced Agricultural Sciences, Zhejiang Agricultural and Forestry University, Hangzhou, China

**Keywords:** UMP kinase, chloroplast development, chlorophyll biosynthesis, gibberellic acid, cellulose synthase, retrograde signaling pathways

## Abstract

The chloroplast is an important cellular organelle and metabolic hub, which is not only responsible for plant photosynthesis but is also involved in the *de novo* biosynthesis of pigments, fatty acids, and hormone metabolisms. Several genes that are responsible for rice leaf color variations have been reported to be directly or indirectly involved in chlorophyll biosynthesis and chloroplast development, whereas a few genes have been functionally confirmed to be responsible for leaf color changes in barley at the molecular level. In this study, we obtained a yellow leaf and dwarf *ygl8* mutant from the progeny of Morex (a variety of barley) seeds treated with EMS. We performed bulked-segregant analysis (BSA) and RNA-seq analysis and targeted a UMP kinase encoding gene, *YGL8*, which generated a splicing site change between exon 5 and 6 of *YGL8* due to a G to A single-nucleotide transition in the 5th exon/intron junction in the *ygl8* mutant. The splicing site change between exon 5 and 6 of *YGL8* had no effects on chloroplast subcellular localization but resulted in an additional loop in the UMP kinase domain, which might disturb the access of the substrates. On one hand, the splicing site change between exon 5 and 6 of *YGL8* downregulated the transcriptional expression of chloroplast-encoded genes and chlorophyll-biosynthesis-related genes in a temperature-dependent manner in the *ygl8* mutant. On the other hand, the downregulation of bioactive GA-biosynthesis*-*related *GA20ox* genes and cell-wall-cellulose-biosynthesis*-*related *CesA* genes was also observed in the *ygl8* mutant, which led to a reduction in plant height. Our study will facilitate the understanding of the regulation of leaf color and plant height in barley.

## Introduction

Photosynthesis, the important process of converting sunlight into chemical energy, provides the basic biochemical substances, such as carbohydrates for plant growth and development, and takes place in the chloroplasts (Nelson and Junge, 2015; Stirbet et al., 2020). Chloroplasts are semi-autonomous organelles and the metabolic centers involved in the synthesis of pigments, such as chlorophyll a and b, nucleotides, amino acids, fatty acids, phytohormones, and many metabolites (Nelson and Junge, 2015; Wang and Grimm, 2015; Daniell et al., 2016; Stirbet et al., 2020). During the course of evolution, most of the chloroplast-encoded genes were transferred into the cell nucleus, and the remaining genes in the chloroplast genome mainly encode proteins for photosynthesis and prokaryotic transcription and translation, including photosynthetic apparatus (Nelson and Junge, 2015), RNA processing factor (matK) (Zoschke et al., 2010; Barthet et al., 2020), RNA polymerase subunits, and ribosomal proteins (Dobrogojski et al., 2020). Thus, the coordination of nuclear-encoded and chloroplast-encoded gene expression is important for chloroplast and plant development (Dobrogojski et al., 2020), and the abnormal development of chloroplasts could decrease photosynthetic efficiency and pigment content, subsequently leading to plant growth retardation and chlorosis (Hein et al., 2009; Zhu et al., 2016; Li et al., 2019; Li et al., 2021).

In rice, more than 100 loci and 40 genes involved in leaf color mutations have been cloned, and the causal genes are mainly directly or indirectly related to chlorophyll biosynthesis and chloroplast development (Chen et al., 2013; Zhou et al., 2013a; Zhou et al., 2013b; Wang et al., 2016; Zhu et al., 2016; Zhou et al., 2017; Chen et al., 2018a; Dong et al., 2019). Although a large number of mutants with full or partial chlorophyll deficiency have been described in barley (Gálová et al., 2000; Prina et al., 2003; Franckowiak and Lundqvist, 2012; Qin et al., 2019), few genes were functionally confirmed at the molecular level due to the relatively late publication of a chromosome-level reference genome (Mascher et al., 2017). The nuclear-encoded *PORB* (protochlorophyllide oxidoreductase), encoding a vital enzyme in the chlorophyll biosynthesis pathway, was truncated by ^60^Co-γ ray treatment in the chlorophyll-deficient barley *NYB* mutant (Liu et al., 2008). The disruption of nuclear-encoded *HvCAO*, which encodes a chlorophyllide oxygenase and is essential for chlorophyll b biosynthesis, resulted in no chlorophyll b formation and a light green leaf phenotype in a barley *fch2* mutant (Mueller et al., 2012). The temperature dependent green-revertible albino leaf of a barley *whs18* mutant was caused by a premature stop codon mutation of nuclear-encoded *HvFLN1* (fructokinase-1-like), which phosphorylates fructose in the carbohydrate metabolism (Qin et al., 2015). Mutations of nuclear-encoded chloroplast-localized HvCMF7 and HvCMF3, members of the CCT (CONSTANS, CONSTANS-like, and TIMING OF CAB1) motif protein family, caused leaf variegation and chlorophyll-deficient phenotypes, respectively, due to retarded chloroplast development that disrupted chloroplast ribosome formation (Li et al., 2019; Li et al., 2021).

Pyrimidine nucleotides are important building blocks of nucleic acids and also function as cofactors for the biosynthesis of a variety of carbohydrates, such as cellulose, hemicellulose, pectin, and secondary metabolites (Kafer et al., 2004). The UMP kinase is a ubiquitous key enzyme for transferring the phosphate group from ATP to UMP to generate UDP, which is subsequently converted to UTP, CTP, and TTP for RNA and DNA synthesis (Charlier et al., 2018; Witte and Herde, 2020). The malfunction of rice *YLG8* (yellow-green leaf 8), which encodes a chloroplast-localized UMP kinase, significantly decreased leaf UDP and UTP content and the transcription and translation of chloroplast-encoded genes, resulting in abnormal chloroplast development, which reduced the chlorophyll content and decreased plant height (Zhu et al., 2016; Chen et al., 2018a; Dong et al., 2019). Additionally, mutation of the *Arabidopsis* chloroplast-localized UMP kinase encoding *Dpt1* (defect in psaA/B transcript accumulation 1) significantly decreased the transcript levels of chloroplast-encoded photosystem I subunits *psaA/B* and impaired chloroplast development, and the mutant failed to grow photoautotrophically and exhibited pale-yellowish leaves and slower plant growth in MS medium supplemented with sucrose (Hein et al., 2009).

Plant height is regulated by a complex and coordinated network involving gibberellic acid (GA) and cellulose biosynthesis (McFarlane et al., 2014; Wang and Wang, 2022). GA, an important plant hormone, plays a vital role in many processes, such as stem or internode elongation (Gupta and Chakrabarty, 2013). Although more than 130 GAs have been discovered, only four types function as bioactive GAs in flowering plants (He et al., 2020). GA20ox, a 2-oxoglutarate-dependent dioxygenase (2-ODD), plays an essential role in bioactive GA biosynthesis (Hedden, 2020; He et al., 2020). The disruption of *GA20ox* genes could result in insufficient bioactive GAs and thus decrease the stem or internode length, ultimately affecting plant height (Sasaki et al., 2002; Paciorek et al., 2022). Cellulose, composed of hydrogen-bonded β-1,4 glucans, is the key component of the cell wall, and its biosynthesis mainly relies on the complexes of cellulose synthase (*CesA*), belonging to the glycosyltranferase-2 (GT-2) superfamily, and CesA-associated proteins (McFarlane et al., 2014). The expression of *CesA* genes could be activated by a GA-mediated signaling pathway (Huang et al., 2015), and the malfunction of *CesA* genes could decrease the plant cellulose content and affect cell wall characteristics, as well as cell size and plant morphologies, such as organ size and plant height (Burn et al., 2002; Wang et al., 2012; Chantreau et al., 2015).

To date, numerous proteins involved in chlorophyll biosynthesis, photosystems, and chloroplast development have been reported to be involved in the regulations of rice leaf color. However, our knowledge of the regulation network of barley leaf color variations is still limited. In the present study, we isolated a yellow leaf mutant, *ygl8*, from the progeny of barley variety Morex seeds treated by EMS and found that the splicing site change between exon 5 and 6 of the chloroplast-localized UMP kinase encoding the *HvGYL8* gene, due to a G to A single-nucleotide transition in the 5th exon/intron junction, caused yellow leaf and a dwarf phenotype in the *ygl8* mutant in a temperature-dependent manner by downregulating chloroplast-encoded genes and the *GA20ox* and *CesA* genes, respectively.

## Materials and methods

### Plant materials and temperature treatments

Barley (*Hordeum vulgare* cv. Morex) seeds were treated with 0.03 mol/L EMS for 16 h, and the yellow leaf phenotype mutant was identified in M2 individuals. The mutant was self-pollinated for another five generations to obtain a relatively stably inherited yellow leaf mutant named *ylg8*. Morex was crossed with the *ygl8* mutant, and F_1_ plants were self-pollinated to generate F_2_ populations for genetic analysis. The greenhouse and field experiments were carried out for hydroponic experiments and BSA and RNA-seq experiments at the Zhejiang Academy of Agricultural Sciences (Hangzhou, Zhejiang Province in China), respectively.

For hydroponic experiments, the barley seeds were sterilized with 10% commercial NaClO, rinsed with tap water, and then germinated in BSM solution (0.5 mM KCl + 0.1 mM CaCl2) for 2 days. Afterward, BSM was changed to 1/5 Hoagland solution with a photoperiod of 14/10 h, light intensity of 200 ± 25 μmol·m−2·s−1, relative humidity of 60%, and constant temperature of 24°C for 8 days (Cai et al., 2022). For different temperature treatments, the seedlings were grown in incubators with 14/10 h light/dark at a constant air temperature of 10°C, 24°C, or 30°C.

### Measurement of chlorophyll a and b and carotenoid contents

Equal fresh weights (approximately 50 mg) of the newly expanded leaves were cut and immersed in 10 ml extract solution (ethanol: acetone: water = 4.5: 4.5: 1) for 16 h in the dark. The homogenates were centrifuged at 3,000 × g for 5 min to remove residual plant debris. The supernatants of these samples were analyzed, and the chlorophyll a and b and carotenoid contents were measured using a spectrophotometer at 663, 645, and 470 nm, and determined using the Lichtenthaler method (Chen et al., 2013).

### DNA and RNA template preparation, library construction, and sequencing

The newest fully expanded leaves of Morex and the *ygl8* mutant at the four-leaf age were collected 1 month after planting in the field and quickly frozen in liquid nitrogen. The total DNA was extracted following the modified hexadecyl trimethylammonium bromide (CTAB) method (Murray and Thompson, 1980). For whole-genome sequencing, the bulked DNA for MutMap analysis was prepared by mixing DNA from 30 *ygl8* individuals in an equal ratio, and the parent DNA for MutMap analysis was prepared by extracting DNA from one Morex sample. A paired-end sequencing library was constructed with an insert size of approximately 350 bp and sequenced on the Illumina Hiseq X Ten platform.

For RNA-seq, the newest fully expanded leaves of Morex and the *ygl8* mutant were collected at the stem elongation stage in the field. Total RNA was isolated using an RNAprep Pure Plant Kit (TIANGEN). RNA-seq library construction was carried out following a method described previously (Shen et al., 2014) and sequenced on the Illumina Hiseq X Ten platform. All sequencing was carried out at Biozeron Biotech, Shanghai.

### MutMap and RNA-Seq analysis

For MutMap analysis, more than 30 depth re-sequencing raw data of bulked pool and parent Morex *ygl8* were filtered using fastp (Chen et al., 2018b) (Version 0.12.4) with the default parameters. The high-quality cleaned reads were aligned to the barley reference genome (Mascher et al., 2021) with BWA (Li and Durbin, 2009) (Version 0.7.17-r1188) using the default parameters. SAMtools (Li et al., 2009) (Version 1.14) was used to convert the mapping results into the sorted and duplication-marked BAM format. SNP detection was performed by BCFtools (Danecek and McCarthy, 2017) (Version 1.14) using the bcftools mpileup command with the parameters ‘–min-MQ 40 –min-BQ 18 –adjust-MQ 50’ and the bcftools call command with the default parameters. The resulting vcf files were filtered using the bcftools filter command with the parameters ‘INFO/MQ>=40 && %QUAL>20’, and the SNP information was extracted using VCFtools (Danecek et al., 2011) (Version 0.1.17). The average SNP-index of the bulked pool of *ygl8* and the parent Morex was estimated via the 2 M sliding window with a 10-kb walking step using Mutplot (Sugihara et al., 2022) (Version 2.3.3) with the parameters ‘–N-bulk 30 –window 2000 –step 10 –min-depth 14 –max-depth 90 –N-rep 10000 –min-SNPindex 0.3’. The genome-wide SNP-index plot was obtained using a custom script written in R (version 4.3.0). The SNP variants were functionally annotated using ANNOVAR (Wang et al., 2010) (Version 2019-10-24).

For RNA-seq analysis, the RNA-seq raw sequencing data of leaf tissues of Morex and the *ygl8* mutant were filtered using fastp (Chen et al., 2018b) (Version 0.12.4) with the default parameters. The high-quality cleaned reads were aligned to the reference genome consisting of the barley reference genome (Mascher et al., 2021) and chloroplast reference genome (Saski et al., 2007) using HISAT2 (Kim et al., 2019). Following alignments, the raw counts for each gene were derived using featureCounts implemented in the R package Rsubread (Liao et al., 2019) and normalized into the number of transcripts per kilobase of exon sequence in a gene per million mapped reads (TPM) using the TMM method (Robinson and Oshlack, 2010). The read alignments of the RNA-seq data to candidate genes were shown in a sashimi plot implemented in the IGV genome browser. The differentially expressed genes in leaf tissues between Morex and the *ygl8* mutant were detected using the DESeq2 method (Love et al., 2014), and the significantly differentially expressed genes were identified with the standard of |log_2_FC| >1 and an adjusted *P* < 0.05. The GO and KEGG enrichment analysis were performed using the R package clusterProfiler (Yu et al., 2012) (Version 4.8.2), and the enrichment results were shown using the R package ggplot2 (Wickham, 2016) (Version 3.4.2).

### Identification of homologous *GA20ox* and *CesA* in barley

The reference barley protein sequences were downloaded from Phytozome (https://phytozome-next.jgi.doe.gov/). Then, *Arabidopsis* GA20ox (AT4G25420) and CesA (AT4G32410) were used as query proteins to carry out a BLASTP search in the barley protein sequences with an E-value < 1e^-10^ as the threshold using DIAMOND (Buchfink et al., 2021) (Version 2.0.11) to identify the barley homologous GA20ox and CesA proteins. Furthermore, the hidden Markov models (HMMs) of the zf-UDP (PF14569.9) and Cellulose_synt (PF03552.17) domains, downloaded from Pfam 35.0 (http://pfam-legacy.xfam.org/), were used to identify the barley homologous CesA proteins using HMMER (http://hmmer.org/) with a “trusted cutoff and E-value < 0.01” as the threshold. The HMMs of the DIOX_N (PF14226.9) and 2OG-FeII_Oxy (PF03171.23) domains were used to identify the barley homologous GA20ox proteins. A total of seven homologous CesA and 24 GA20ox were identified in barley.

### Construction of vectors

Primers were designed according to the open reading frames of the barley *YGL8* and *OsYSS1* (Zhou et al., 2017) genes. The corresponding sequences were amplified from the cDNA of barley (Morex or *ygl8*) or rice and cloned into the pMD18T vector (Takara). The clones were selected by PCR and sequenced for confirmation. For the subcellular localization of YGL8 and ygl8 in tobacco leaves, seamless cloning was used to introduce the *YGL8* or *ygl8* genes, fused to the *GFP* gene amplified from the pH7WGF2.0 vector, into the pCAMBIA1300 vector, after the 35S promoter amplified from the pH7WGF2.0 vector in advance, to obtain YGL8-GFP or ygl8-GFP. Additionally, seamless cloning was used to introduce the *OsYSS1* gene, fused to the *mCherry* gene amplified from pSAT4a-mCherry-N1, into a modified pCAMBIA1300 vector to obtain OsYSS1-mCherry. The primer information is listed in **Supplementary Table 1**.

### Subcellular localization of YGL8 in tobacco leaves

The subcellular localization vectors of *YGL8* were transiently expressed in tobacco (*Nicotiana benthamiana*) leaves by *Agrobacterium*-mediated infiltration. OsYSS1-meCherry was used as a chloroplast marker. The *Agrobacterium* strain C58C1 harboring p19 was used to prevent the onset of post-transcriptional gene silencing (PTGS) in the infiltrated leaves. Infiltrated tobacco plants were grown for another 3 days for GFP and mCherry imaging using a Zeiss LSM710NLO confocal laser-scanning microscope. The excitation/emission wavelengths were 488 nm for GFP and 561 nm for mCherry.

### Protein structure prediction and visualization

The protein structures of YGL8 and ygl8 were predicted using SWISS-MODEL (https://swissmodel.expasy.org/) with the default parameters. The resulting pdb files of the corresponding proteins were compared and shown using PyMOL (DeLano, 2002).

### Quantitative real-time PCR

Total RNA was extracted from the newest fully expanded leaves of Morex and the *ygl8* mutant at 2 weeks in the hydroponic experiment or stem elongation stage in the field experiment using an RNAprep Pure Plant Kit (Tiangen Co. Ltd., China). First-strand cDNAs were synthesized using a Fasting RT Kit with gDNase (Tiangen Co. Ltd., China). qRT-PCR was performed using the Talent qPCR PreMix (Tiangen Co. Ltd., China), and the barley actin gene was used as the endogenous control. Reactions containing SYBR premix were carried out in final volumes of 20 ul containing 0.3 umol of the appropriate primers and 2 * PCR master mix. The 2^-△△t^ method was used to calculate the relative levels of gene expression. The primers used for qRT-PCR are listed in **Supplementary Table 1**.

## Results

### Phenotype of the *ygl8* mutant

**Figure 1:**
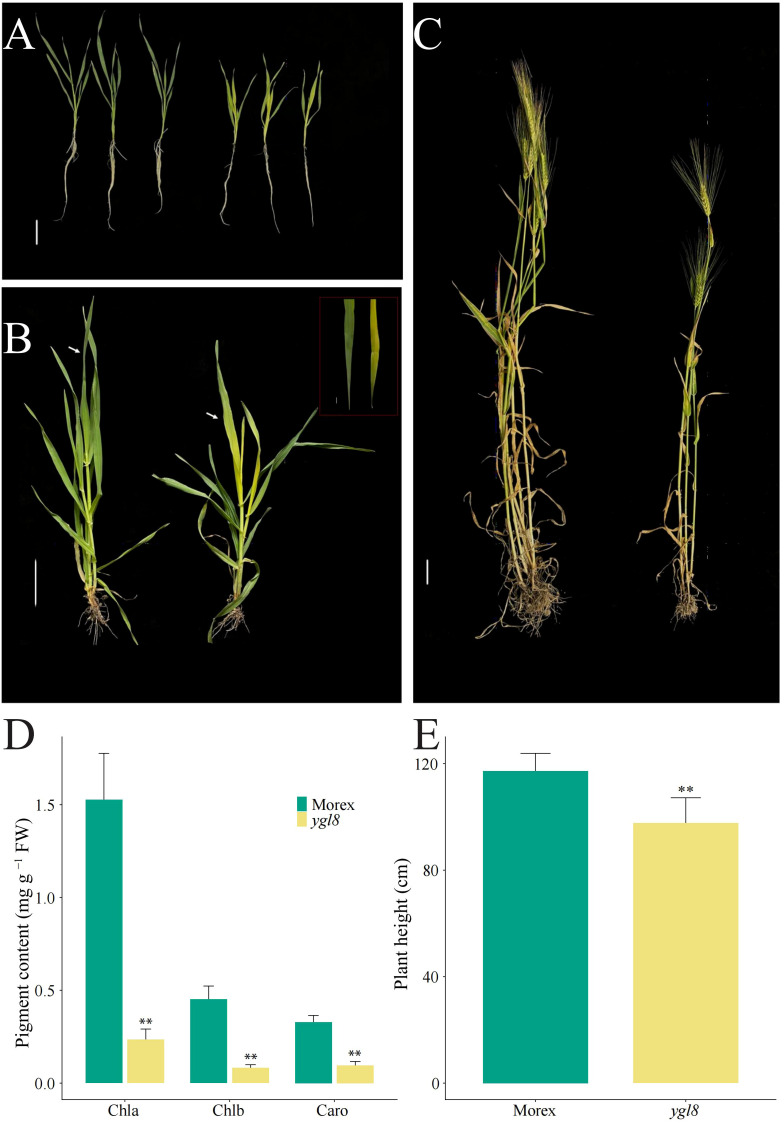
The yellow leaf *ygl8* mutant shows significantly decreased pigment content and plant height. The phenotype of Morex (on the left) and the *ygl8* mutant (on the right) at the 21-day seedling stage in the greenhouse **(A)**, and at the stem elongation stage **(B)** and mature stage in the field **(C)**. The white arrows indicate the newly merged leaves. **(D, E)** The pigment content **(D)** and plant heights **(E)** of Morex and the *ygl8* mutant at the 21-day seeding stage in the greenhouse and at the mature stage in the field. Chla, Chlb, and Caro represent chlorophyll a, chlorophyll b and carotenoid, respectively. Scale bars: 5 cm. Data are mean + SD (n = 6) for **(D, E)**. Significant differences between Morex and *ygl8* mutant are indicated (**P < 0.01; Student’s *t* test).

We isolated a yellow leaf mutant, *ygl8*, from the progeny of barley variety Morex seeds treated with EMS, and the newly emerged leaves of the *ygl8* mutant were yellowish throughout the vegetative growth period (**Figures 1A, B**). To investigate whether the pigment contents changed in the yellow leaf of the *ygl8* mutant, we detected the pigment contents of the newest fully expanded leaves of Morex and the *ylg8* mutant grown in hydroponic solution for 3 weeks and observed significantly decreased pigment contents in the *ygl8* mutant compared with Morex (**Figure 1D**). The chlorophyll a and b and carotenoid contents of the *ygl8* mutant decreased 15%, 18%, and 29%, respectively, compared with those of Morex (**Figure 1D**). Additionally, we observed that the plant height of the *ygl8* mutant decreased to 83% of that of Morex at the mature stage in the field (**Figures 1C, E**). These results suggest that pigment synthesis in the leaves of the *ygl8* mutant was dramatically inhibited.

### Identification of the gene associated with the *ygl8* mutant

For the rapid identification of the gene responsible for the yellow leaf phenotype in the *ygl8* mutant, the bulked DNA pool, consisting of 30 randomly selected *ygl8* individuals with a yellowish leaf phenotype, and Morex were subjected to whole-genome sequencing for SNP detection. In total, we obtained 1,397,745 M and 1,002,230 M clean reads for the Morex and *ylg8* bulked pools, respectively (**Supplementary Table 2**). These reads were aligned to the Morex reference genome (Mascher et al., 2021) and 953,254 SNPs were detected.

For each SNP position, the value of the SNP-index was derived, and the ΔSNP-index values (the difference of the SNP-index of Morex and the bulked *ygl8* pool) were plotted against each SNP position on the seven chromosomes (**Supplementary Figure 1**). Furthermore, 4,312 SNP positions with an ΔSNP-index = 1 were identified on seven chromosomes and functionally annotated according to genetic variant types. Then, we observed 130 SNP positions with an ΔSNP-index = 1 in genic regions, including 2 kb upstream and downstream, and the related 129 genes were focused on as candidate genes for the yellow leaf phenotype in the *ygl8* mutant (**Supplementary Table 3**). Moreover, we compared gene expression in the leaf tissues of Morex and the *ygl8* mutant at the stem elongation stage in the field using RNA-seq and found that 59 out of the 129 candidate genes were expressed in the leaf tissues (**Supplementary Table 3**).

**Figure 2:**
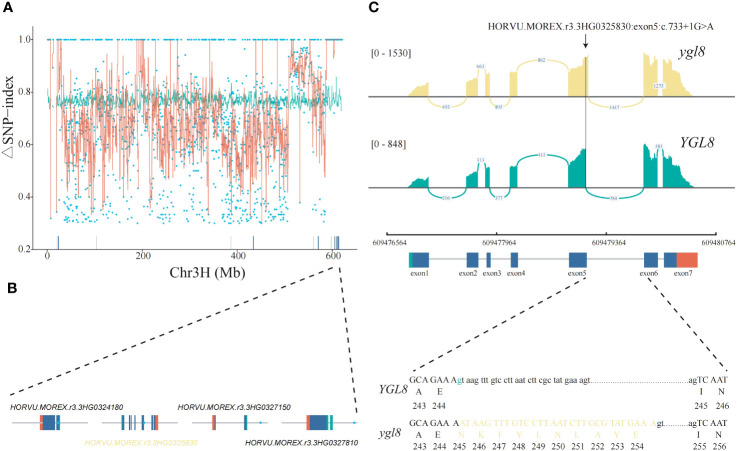
Identification of the candidate gene responsible for the yellow leaf phenotype of the *ygl8* mutant by Mutmap and RNA-seq. **(A)** ΔSNP-index plot of the barley chromosome 3H generated by Mutmap analysis. The red lines correspond to the sliding window average values of the ΔSNP-index of 2-Mb intervals with a 10-Kb increment. The blue points represent SNPs identified between Morex and the *ygl8* mutant. The green lines show the 99% confidence limit of the ΔSNP-index value under the null hypothesis. The blue and gray bars above the x axis demonstrate the expressed and unexpressed genes in the leaf RNA-seq data, respectively. **(B)** The gene structures and mutation positions of the four candidate genes expressed in leaves. The blue points represent the positions with an ΔSNP-index = 1. The green, blue, and red boxes indicate the 5′ UTR, CDS, and 3′ UTR, respectively. **(C)** The splicing site change between exon 5 and 6 of *HORVU.MOREX.r3.3HG0325830* in the *ylg8* mutant due to a G to A single-nucleotide transition at the 5th exon/intron junction. The SNP location impacting the splicing site change between exon 5 and 6 of *YGL8* in the *ygl8* mutant is highlighted with a black arrow. The read alignments are shown in a sashimi plot implemented in the IGV genome browser. Green marks the G to A transition. The exon and intron sequences are indicated in upper and lower cases, respectively. Yellow denotes the added sequences in the exon of the *ygl8* gene for the splicing site change between exon 5 and 6.

To alleviate the effects of large variations of single ΔSNP-index values, the sliding window average values of the ΔSNP-index were calculated, and we further narrowed down the candidate genomic region to the chromosome 3H: 604,680,000-622,110,000, comprehensively considering the genetic variant types, gene functional annotations, and gene expression (**Figure 2A**; **Supplementary Table 3**). There were seven genes with an ΔSNP-index =1 in this region but only four were expressed in our RNA-seq data (**Figure 2B**). Based on the genetic variant types and functional annotations, we finally focused on *HORVU.MOREX.r3.3HG0325830* (named *YGL8*), which encoded one UMP kinase and contained one G to A single-nucleotide transition in the 5th exon/intron junction in the *ygl8* mutant; the transition might lead to a splicing site change between exon 5 and 6 of *YGL8* in the *ygl8* mutant. Notably, the mutations in the *Arabidopsis* and rice orthologous *YGL8* genes were previously reported to affect chloroplast development, and thus resulted in a low chlorophyll content and yellowish leaf phenotype (Hein et al., 2009; Zhu et al., 2016; Chen et al., 2018a; Dong et al., 2019). To confirm the splicing site change between exon 5 and 6 of the *YGL8* gene in the *ygl8* mutant, we investigated the read alignments around the *YGL8* gene using the RNA-seq data of Morex and the *ygl8* mutant and observed the existence of the splicing site change between exon 5 and 6 of *YGL8* in the *ygl8* mutant (**Figure 2C**). Furthermore, Sanger sequencing revealed that the splicing site change between exon 5 and 6 of *YGL8* resulted in a 30-bp insertion in the 5th exon in the *ygl8* mutant (**Figure 2C**).

To investigate whether the yellow leaf phenotype of the *ygl8* mutant was controlled by a single gene, we crossed Morex and the *ygl8* mutant to obtain F1 progeny, and the F1 plants were self-pollinated to generate F2 progeny. All F1 progeny exhibited the normal green leaf phenotype, and the leaf phenotype of the F2 plants segregated in a 3:1 ratio (150 green: 40 yellow), which indicated that the yellow leaf phenotype of the *ygl8* mutant was conferred by a single recessive mutation. These results reveal that the yellow leaf phenotype of the *ygl8* mutant might be the result of the splicing site change between exon 5 and 6 of *YGL8* due to a G to A single-nucleotide transition in the 5th exon/intron junction.

### The chloroplast subcellular localization of *YGL8* is not affected by the splicing site change between exon 5 and 6

**Figure 3:**
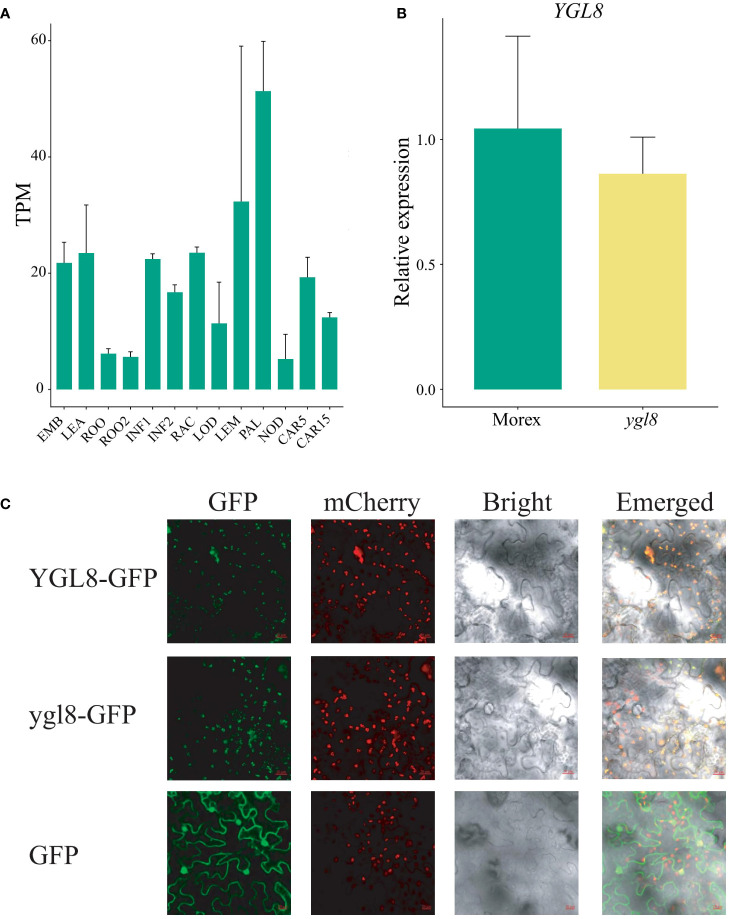
The splicing site change between exon 5 and 6 of *YGL8* does not affect the gene transcript level and chloroplast subcellular localization. **(A)** The *YGL8* gene transcript level in the published different developmental tissues (Mascher et al., 2017). EMB, embryo 4 days after planting (dap); LEA, leaf 17 dap; ROO, 17 dap root; ROO2, root 28 dap; INF1, inflorescences 30 dap; INF2, inflorescences 50 dap; RAC, rachis 35 dap inflorescence; LOD, lodicule 42 dap; LEM, lemma 42 dap; PAL, palea 42 dap; NOD, the third stem internode (42 dap); CAR5, caryopsis 5 days post-anthesis (dpa); CAR15, caryopsis 15 dpa. Data are means + SD (n=3). **(B)** Transcript level of *YGL8* in the leaves of Morex and the *ygl8* mutant grown in hydroponic culture for 3 weeks. **(C)** The subcellular localization of YGL8 and ygl8 in tobacco leaves. The GFP were fused to the C terminus of YGL8 (YGL8-GFP) and ylg8 (ylg8-GFP). OsYSS1-mCherry was used as a chloroplast protein marker. The 35S::GFP vector (GFP) was used as a control. The fluorescence signals in tobacco leaves were detected with a confocal laser-scanning microscope. Scale bars: 20 um.

To comprehensively understand the transcriptional expression patterns of *YGL8*, the RNA-seq data from different developmental tissues of Morex were derived (Mascher et al., 2017), and the analysis demonstrated that *YGL8* was constitutively expressed, with a relatively higher expression level in photosynthetic tissues (**Figure 3A**). To determine the influences of the splicing site change between exon 5 and 6 on the transcriptional level of *YGL8*, we examined the transcripts of *YGL8* in the 3-week-old leaf tissues of Morex and the *ygl8* mutant grown in hydroponic solution, and no significant transcriptional changes were observed (**Figure 3B**).

To investigate whether the splicing site change between exon 5 and 6 of *YGL8* affected the subcellular localization, the YGL8 or ygl8 fused with GFP (YGL8-GFP or ygl8-GFP) were transiently expressed in tobacco leaves. We observed strong GFP signals of YGL8 and ygl8 in the chloroplast, which coincided with the chloroplast marker signals of OsYSS1-mCherry (Zhou et al., 2017) (**Figure 3C**). These results demonstrate that the splicing site change between exon 5 and 6 of *YGL8* has no effect on the gene transcript and chloroplast subcellular localization.

### The splicing site change between exon 5 and 6 of *YGL8* affects protein structure

**Figure 4:**
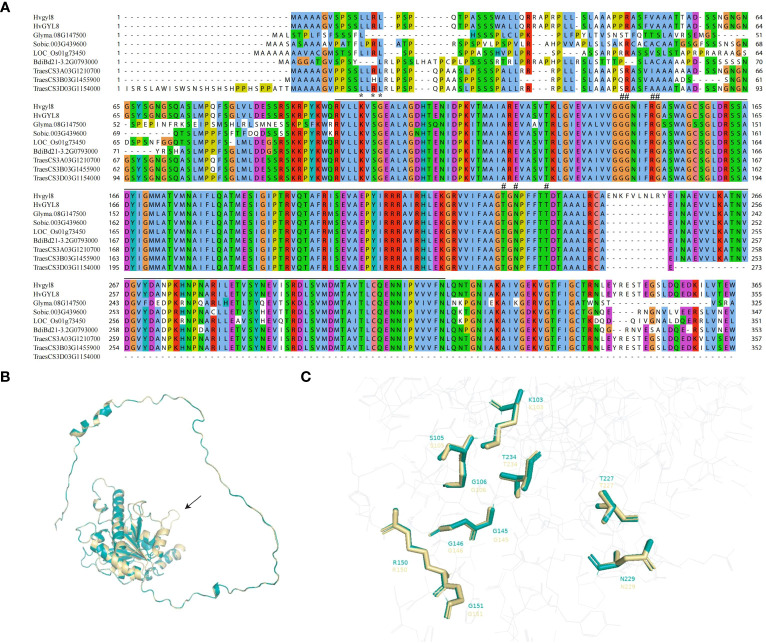
Amino acid alignments of orthologous YGL8 proteins of different plant species and protein structure comparison of barley YGL8 and ygl8. **(A)** The amino acid sequence alignments of barley YGL8 and ygl8 and the orthologs of soybean, *Sorghum*, rice, *Brachypodium distachyon*, and wheat. The black lines indicate the conserved UMP kinase domain. # indicates residues known to interact with UMP. # indicates residues known to interact with either UDP or UTP (Hein et al., 2009). **(B)** The predicted three-dimensional protein structure comparison of barley YGL8 and ygl8. The black arrow indicates the loop composed of the added 10 amino acids due to the splicing site change between exon 5 and 6 of *YGL8*. **(C)** Enlarged image of conformations of the essential amino acids, previously reported to be responsible for substrate binding. The numbers indicate the amino acid positions in the corresponding proteins. The model was constructed according to SWISS-MODEL (https://swissmodel.expasy.org/). The structures are shown using PyMOL. For **(B, C)**, green and yellow represent the barley YGL8 and ygl8 proteins, respectively.

We compared the YGL8 orthologous proteins of different species and observed high similarity among them, especially in the UMP kinase domain (**Figure 4A**). Given that the splicing site change between exon 5 and 6 of *YGL8* was located in the UMP kinase domain (**Figure 4A**), we assumed that the mutation might affect the protein structure of the conserved UMP kinase domain. To verify this hypothesis, we predicted and compared the protein structures of YGL8 with ygl8 and found that the splicing site change between exon 5 and 6 of *YGL8* resulted in an additional loop in the UMP kinase domain structure in the ygl8 protein (**Figure 4B**). Furthermore, the ten key amino acids, previously reported to be important for interactions with the substrates UMP or UTP (Hein et al., 2009), were examined, and we only observed slight conformational changes for five of the ten amino acids in the predicted ylg8 protein structure (**Figure 4C**; **Supplementary Figure 3**). These results suggest that the newly generated loop in ygl8 protein did not significantly affect the UPM domain structure, whereas the additional loop might disturb the access of the substrates to the kinase domain, thus resulting in a full or partial malfunction of the *YGL8* gene.

### Enrichment analysis of differentially expressed genes in the leaf tissues of the *ygl8* mutant

**Figure 5:**
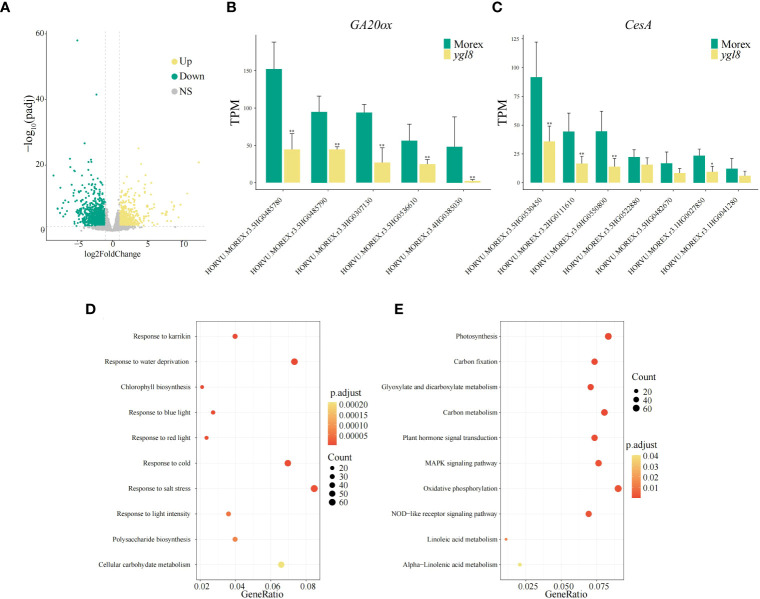
Transcriptome analysis of the leaf tissues of Morex and the *ygl8* mutant. **(A)** Volcano map of differentially expressed genes (DEGs) between Morex and the *ygl8* mutant. Up, Down and NS indicate upregulation, downregulation, and no significant changes of gene expression in the *ygl8* mutant compared with Morex. **(B, C)** Transcript levels of differentially expressed *GA20ox* **(B)** and *CesA* **(C)** homologous genes. Data are mean + SD (n=3) for **(B, C)**. Significant differences between Morex and the *ygl8* mutant are indicated (*P.adj < 0.05, **P.adj < 0.01). **(D)** Top 10 significantly enriched GO terms of DEGs. **(E)** Top 10 significantly enriched KEGG terms of DEGs. The terms are sorted by p.adjust values in **(D, E)**.

To clarify which genes are affected in terms of transcription levels due to the splicing site change between exon 5 and 6 of *YGL8*, we investigated the transcriptomes of the leaf tissues of Morex and the *ygl8* mutant. Compared with Morex, we observed 2,401 significantly differentially expressed nuclear-encoded genes, including 817 upregulated genes and 1,584 downregulated genes (**Figure 5A**; **Supplementary Table 4**). The most significantly enriched GO terms included response to karrikin, chlorophyll biosynthetic process and saccharide biosynthetic process, and response to abiotic stresses such as cold stress and light intensity (**Figure 5D**; **Supplementary Table 6**). The most significantly enriched KEGG pathways included photosynthesis, carbon fixation and metabolism, plant hormone signal transduction and MARK signaling pathway, and linoleic acid metabolism (**Figure 5E**; **Supplementary Table 7**). These results suggest that photosynthesis and plant signaling transduction are affected by the splicing site change between exon 5 and 6 of the *YGL8* gene in the *ygl8* mutant.

### The *GA20ox* and *CesA* genes are downregulated in the *ygl8* mutant

Bioactive GAs are vital endogenous plant growth regulators, which affect stem and internode elongation partially by influencing the cell wall characteristics via regulating *CesA* genes (Gupta and Chakrabarty, 2013; Huang et al., 2015). Among the differentially expressed genes in the RNA-seq data from Morex and the *ygl8* mutant, we observed that five homologous *GA20ox* genes and all seven homologous *CesA* genes were significantly downregulated in the *ygl8* mutant (**Figures 5B, C**; **Supplementary Table 8**). Furthermore, we examined the gene expression in all 24 homologous *GA20ox* genes in the leaf and internode tissues from a previous published paper (Mascher et al., 2017) and found that the differentially expressed *GA20ox* genes in our RNA-seq data had the highest expression levels of all the homologous members in the internode tissues (**Supplementary Figure 4**; **Supplementary Table 9**). These results demonstrate that the splicing site change between exon 5 and 6 of *YGL8* downregulates the expression of the *GA20ox* and *CesA* genes in the *ygl8* mutant and thus leads to the dwarf phenotype.

### *YGL8* is temperature sensitive and affects the expression of chloroplast-encoded and chlorophyll synthetic genes

**Figure 6:**
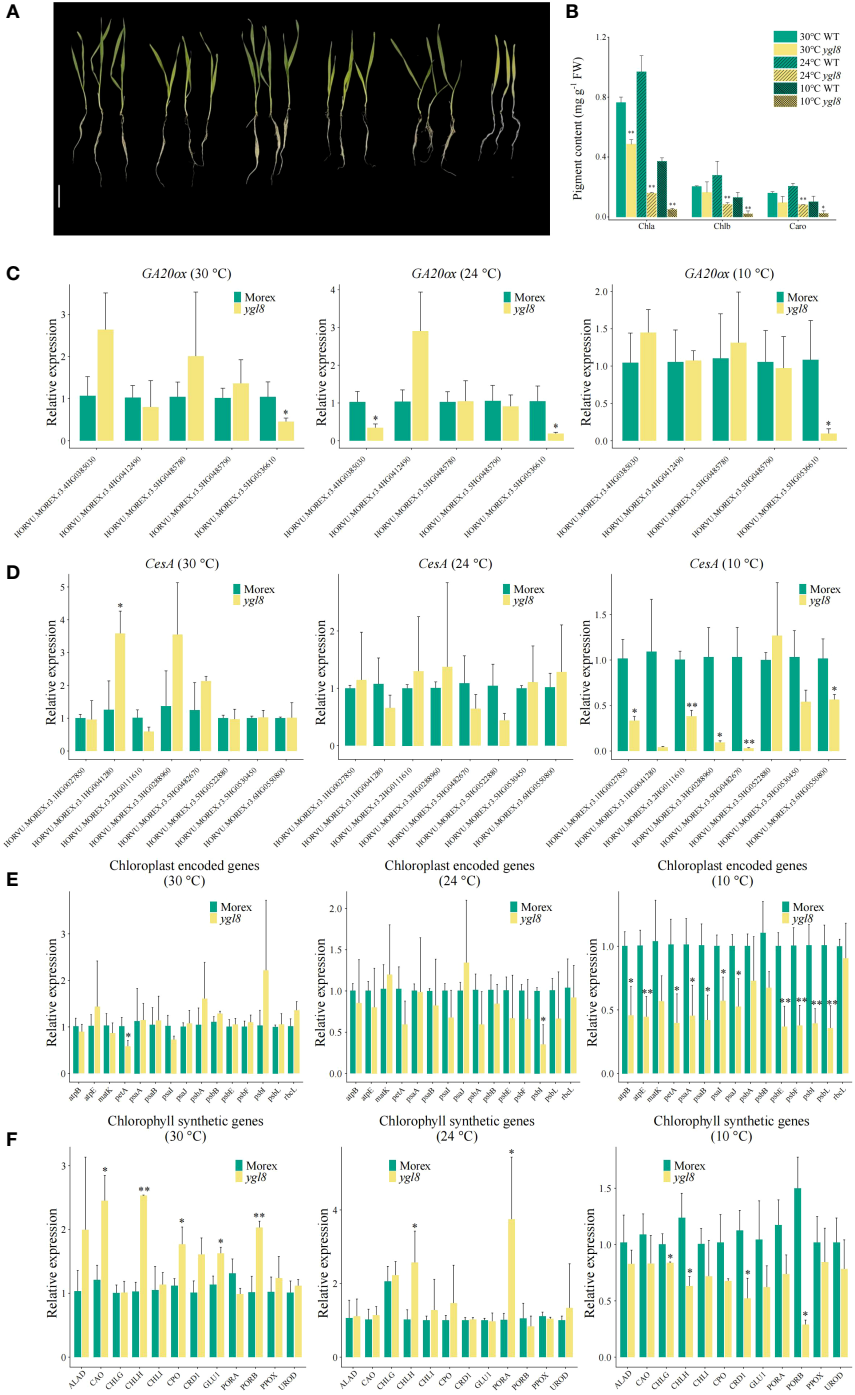
The effects of the splicing site change between exon 5 and 6 of *YGL8* are dependent on temperature. **(A)** The phenotypes of Morex and the *ygl8* mutant grown in different temperatures for 2 weeks. Scale bar: 5 cm. **(B)** The pigment contents of the leaves of Morex and the *ygl8* mutant under different temperatures. **(C–F)** The transcript levels of *GA20ox* **(C)**, *CesA* **(D)**, chloroplast-encoded **(E)**, and chlorophyll-biosynthesis-related genes **(F)** in the leaf tissues of Morex and the *ygl8* mutant at different temperatures. Data are mean + SD (n=3). Significant differences between Morex and the *ygl8* mutant are indicated (*P < 0.05, **P < 0.01; Student’s *t* test).

Many genes responsible for leaf color variations have been reported to be temperature sensitive (Chen et al., 2013; Shi et al., 2015; Liu et al., 2020). To address whether the yellow leaf phenotype of the *ygl8* mutant was dependent on the temperature conditions, we grew Morex and the *ylg8* mutant in a hydroponic solution under different temperature conditions. Although a more yellowish leaf of the *ygl8* mutant was observed at a low temperature (10°C) than at a normal culture temperature (24°C), the newly emerged leaf of the *ygl8* mutant was green at a high temperature (30°C) (**Figure 6A**), which was consistent with the pigment content of the *ygl8* mutant (**Figure 6B**). Furthermore, we examined the transcript levels of the *GA20ox* and *CesA* genes, which showed differential expression in our RNA-seq data, in the leaf tissues of Morex and the *ygl8* mutant under different temperature conditions. The downregulation of *CesA* genes in the *ygl8* mutant was only observed at a low temperature and no significant transcript changes were observed at normal or high temperatures (**Figure 6D**). Interestingly, we did not observe obvious transcript changes of *GA20ox* genes under different temperature conditions in the *ygl8* mutant compared with Morex (**Figure 6C**), inconsistent with our RNA-Seq data (**Figure 5B**), which might be caused by different developmental sampling stages. Additionally, different conclusions about affected genes have been obtained in the rice *ygl8* mutant for different sampling timings (Zhu et al., 2016; Chen et al., 2018a; Dong et al., 2019).

Given that the disruption of chloroplast-localized UMP kinase affected chloroplast-encoded gene expression and resulted in abnormal chloroplast development, subsequently resulting in a chlorophyll-deficient leaf phenotype in *Arabidopsis* and rice (Hein et al., 2009; Zhu et al., 2016; Chen et al., 2018a; Dong et al., 2019), we examined the chloroplast-encoded and chlorophyll synthetic genes in the *ygl8* mutant under different temperature conditions. We found that the chloroplast thylakoid membrane major component encoding genes, including photosystem complex subunit encoding genes (*psaA*, *psaB*, *psaI*, *psaJ, psbE, psbF, psbI*, and *psbL)* (Nelson and Junge, 2015), ATP synthase subunit encoding genes (*atpB* and *atpE*) (Dobrogojski et al., 2020), and the cytochrome b_6_/f subunit encoding gene *petA* (Dobrogojski et al., 2020), showed similar expression patterns in the *ygl8* mutant to those in Morex, with downregulation at low temperatures and no significant changes at normal or high temperatures (**Figure 6E**). Similarly, several chlorophyll synthetic genes were only downregulated in the *ygl8* mutant compared with Morex at low temperatures (**Figure 6F**). These results suggest that the yellow leaf phenotype of the *ygl8* mutant is temperature dependent.

## Discussion

The chloroplast, as the metabolic hub of a cell, is also the site of hormone metabolism, such as GA and jasmonic acid, and the *de novo* biosynthesis of pigments such as chlorophyll a and b and abnormal development of chloroplasts could influence the expression of photosynthesis-associated nuclear genes (*PhANGs*) and pigment production (de Souza et al., 2017; Jan et al., 2022). In this study, we identified a yellow leaf and dwarf *ygl8* mutant and found that these phenotypes were caused by a splicing site change between exon 5 and 6 of a UMP kinase encoding *YGL8*, due to a G to A single-nucleotide transition in the 5th exon/intron junction. The splicing site change between exon 5 and 6 of *YGL8* had no effect on the chloroplast subcellular localization (**Figures 3B, C**) but generated an additional loop in the UMP kinase domain (**Figure 4B**), which might affect the enzyme function by disturbing the access of substrates and subsequently decreasing chloroplast UDP and UTP contents and impairing DNA and RNA synthesis, consistent with the downregulation of chloroplast-encoded gene expression (**Figure 6E**). Our RNA-seq results and hydroponic experiments demonstrated that the yellow leaf and plant dwarfism of the *ygl8* mutant is caused by the downregulation of chloroplast-encoded genes and *GA20ox* and *CesA* genes, respectively (**Figures 5B, C**, **6C–E**), and the effects of the splicing site change between exon 5 and 6 of *YGL8* on the phenotype and gene expression are temperature dependent (**Figure 6**; **Supplementary Figure 5**).

A few yellow leaf barley mutants have been reported previously (Qin et al., 2015; Wang et al., 2017; Qin et al., 2019; Gajecka et al., 2021). One thermo-inducible chlorophyll-deficient mutant, named V-V-Y, was previously identified from gamma-radiated barley variety Vlamingh seeds, and the *vvy* mutant showed green leaf at a normal temperature but turned yellowish at temperatures above 30°C (Wang et al., 2017), which was different from the temperature-dependent manner of the *ygl8* mutant in this study (**Figure 6**). In addition, the gene for the *vvy* mutant was mapped to the long arm of chromosome 4H (Wang et al., 2017); however, the *YGL8* gene identified in this study is located on chromosome 3H (**Figures 2A, B**; **Supplementary Table 3**). *whs18*, a natural barley mutant, showed abnormal chloroplast development and stage green-revertible albino under the field condition, and the temperature under the ice-point during growth was necessary for leaf color variations (Qin et al., 2015), which was also different from the temperature-dependent manner of the *ygl8* mutant in this study given the observation of a yellow leaf phenotype under constant 24°C or 10°C conditions (**Figures 1A**, **6A**). Mutation of the plastid- and nucleus-localized *HvCMF3* gene, a member of the CCT motif protein family, led to a yellowish/light green leaf color due to reduced ribosome accumulation and impaired grana organization and chloroplast development (Li et al., 2021). Another yellow leaf color mutant, *ygl*, obtained from the progeny of barley cultivar Edamai 934 seeds treated with EMS, showed impaired chloroplast ultrastructure, yellow leaf, and a decreased plant height phenotype. The QTL controlling leaf color phenotype of the *ygl* mutant was initially mapped in a 12.7 cM interval on the end of chromosome 3H (50.7082–52.0299 cM), but no specific gene responsible for the phenotype was confirmed using the strategy of BSA based on SSR markers and a contig level Morex reference genome (Qin et al., 2019). In this study, we carried out BSA based on high-throughput sequencing and the updated chromosome level Morex reference genome (Mascher et al., 2021) and targeted the *YGL8* gene on the end of chromosome 3H (604,680,000–622,110,000 bp). Considering the similar phenotypes and chromosome positions, there is a possibility that *YGL8* is the causal gene for the phenotype of the previously reported *ygl* mutant; however, this needs further confirmation.

The chloroplast can act as an environment sensor and communicate with the nucleus through retrograde signaling pathways to regulate nuclear gene expression in response to developmental cues and stress that affect photosynthesis and growth (Nott et al., 2006; de Souza et al., 2017; Jan et al., 2022). Four distinct retrograde signaling pathways have been recognized in higher vascular plants based on the sources of the signals: the accumulation of tetrapyrrole intermediates such as Mg-protoporphyrin IX, the inhibition of plastid gene expression, changes in plastid redox status, and the production of ROS (Nott et al., 2006; de Souza et al., 2017; Jan et al., 2022). The disruption in retrograde signaling pathways could result in the sustained expression of *PhANGs*, such as the light-harvesting chlorophyll a/b-binding (LHC) protein encoding gene *LHCB1*, even under stress conditions, such as photooxidative stress and ABA treatment (Martinez et al., 2003; Koussevitzky et al., 2007; de Souza et al., 2017). Based on the overproduction of ROS, such as H_2_O_2_, in leaf tissues in the rice orthologous *YGL8* mutant, we expected the transcript levels of *LHC* genes to be reduced in the *ygl8* mutant compared with Morex. Interestingly, we observed the upregulation of many *LHC* genes in leaf tissues of the *ygl8* mutant compared with Morex in our RNA-seq data (**Supplementary Figure 5A**; **Supplementary Table 10**), and a similar expression pattern was also observed at low temperatures in hydroponic experiments (**Supplementary Figure 5B**), demonstrating that the retrograde signaling transduction was disrupted in the *ygl8* mutant. Chlorophyll and heme share a common precursor of tetrapyrrole biosynthesis, and at the branchpoint, Mg^2+^ and Fe^2+^ are inserted into protoporphyrin IX by Mg-chelatase and ferrochelatase (FC) to subsequently produce chlorophyll and heme, respectively (Jan et al., 2022). The overaccumulation of heme could feedback inhibit the tetrapyrrole biosynthesis pathway (de Souza et al., 2017; Jan et al., 2022) and thus reduce the production of retrograde signaling Mg-protoporphyrin IX, resulting in the sustained expression of the *LHCB* gene independent of chloroplast status (Martinez et al., 2003). Based on the upregulation of *FC1* in the *ygl8* mutant at low temperatures in the hydroponic experiment (**Supplementary Figure 5B**), we speculated that cold-stress-induced ROS decreased *LHC* gene expression in Morex, whereas the accumulation of heme via increasing *FC1* gene expression in the *ygl8* mutant partially alleviated the downregulation of the *LHC* genes, eventually resulting in an upregulation of *LHC* in the *ygl8* mutant compared with Morex (**Supplementary Figure 5B**). However, other complex mechanisms might be involved in the regulation of *LHC* gene expression in the *ygl8* mutant. In summary, this study facilitates our understanding of the barley leaf color and plant height regulation mechanism, and the *ygl8* mutant is a valuable tool for investigating the retrograde signaling pathway in barley in the future.

## Conclusion

In this study, we identified a yellow leaf and dwarf *ygl8* mutant from the progeny of Morex seeds treated with EMS, and the phenotype of the *ygl8* mutant was caused by a splicing site change between exon 5 and 6 of *YGL8*, which encodes a chloroplast-localized UMP kinase, due to a G to A single-nucleotide transition in the 5th exon/intron junction. The splicing site change between exon 5 and 6 of *YGL8* resulted in the insertion of ten amino acids in the UMP kinase domain and might impair enzyme function by disturbing the access of the substrates. The yellow leaf phenotype of the *ygl8* mutant was caused by the downregulation of chloroplast-encoded genes and chlorophyll-biosynthesis-related genes in a temperature-dependent manner. For the decreased plant height phenotype of the *ygl8* mutant, the downregulation of *GA20ox* genes, which are responsible for bioactive GA biosynthesis, and *CesA* genes, which are responsible for cell wall cellulose biosynthesis, relative to Morex was observed. Our study will facilitate the understanding of leaf color and plant height regulation in barley.

## Data availability statement

The data presented in the study are deposited in the NCBI repository, accession number PRJNA1027084.

## Author contributions

XX: Data curation, Investigation, Writing – original draft. LL: Investigation, Writing – review & editing. KC: Investigation, Writing – review & editing. XS: Investigation, Writing – review & editing. WY: Data curation, Formal analysis, Software, Writing – original draft, Writing – review & editing. JW: Conceptualization, Formal analysis, Funding acquisition, Resources, Supervision, Writing – original draft, Writing – review & editing.
