# Supplementary figures and images for: A splicing site change between exon 5 and 6 of the nuclear-encoded chloroplast-localized HvYGL8 gene results in reduced chlorophyll content and plant height in barley
Source: Front Plant Sci. 2023 Dec 12;14:1327246. doi: 10.3389/fpls.2023.1327246 (PMC10773589; doi:10.3389/fpls.2023.1327246)

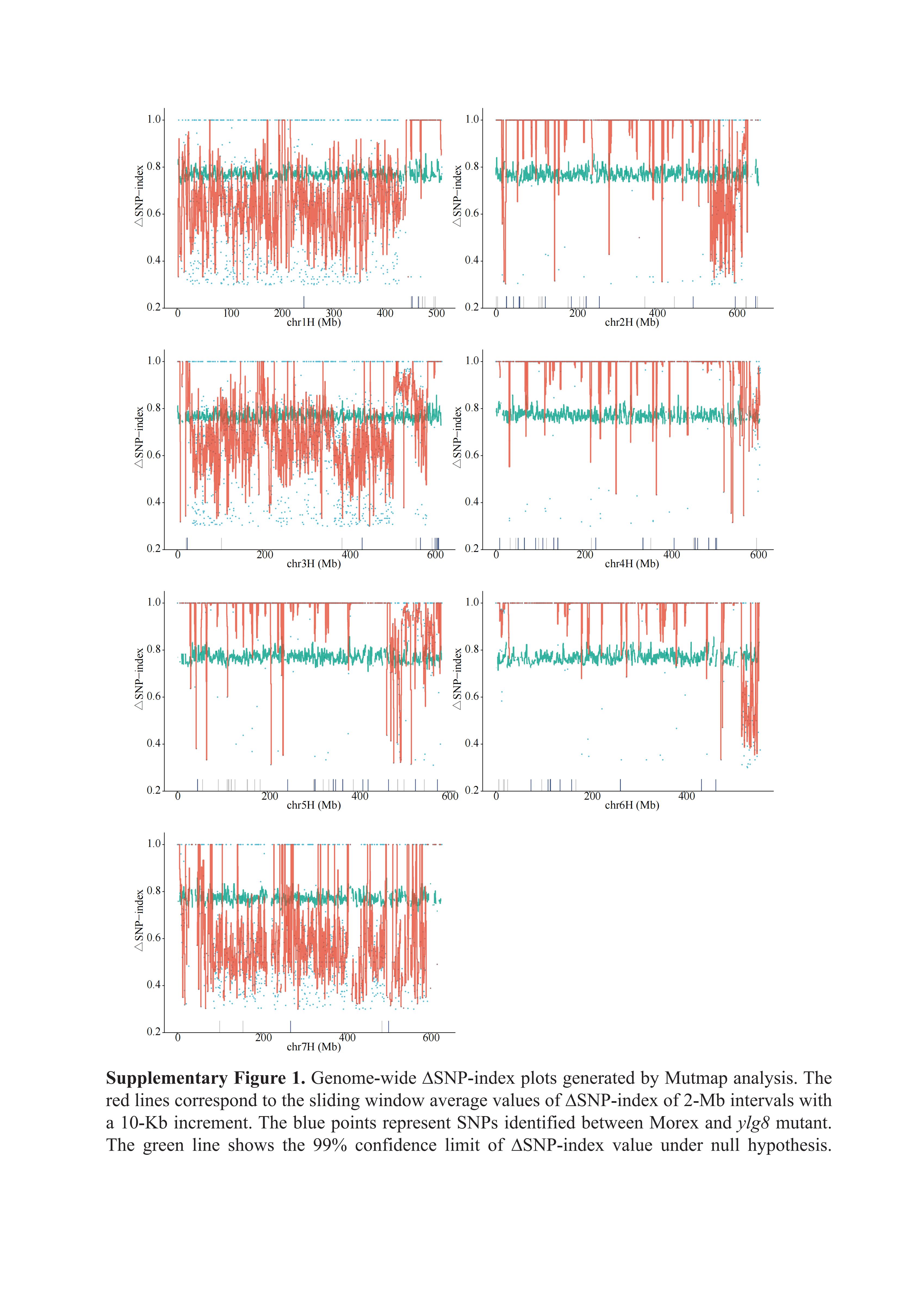

Supplement: Supplementary file 2 [file Image_1.jpeg]

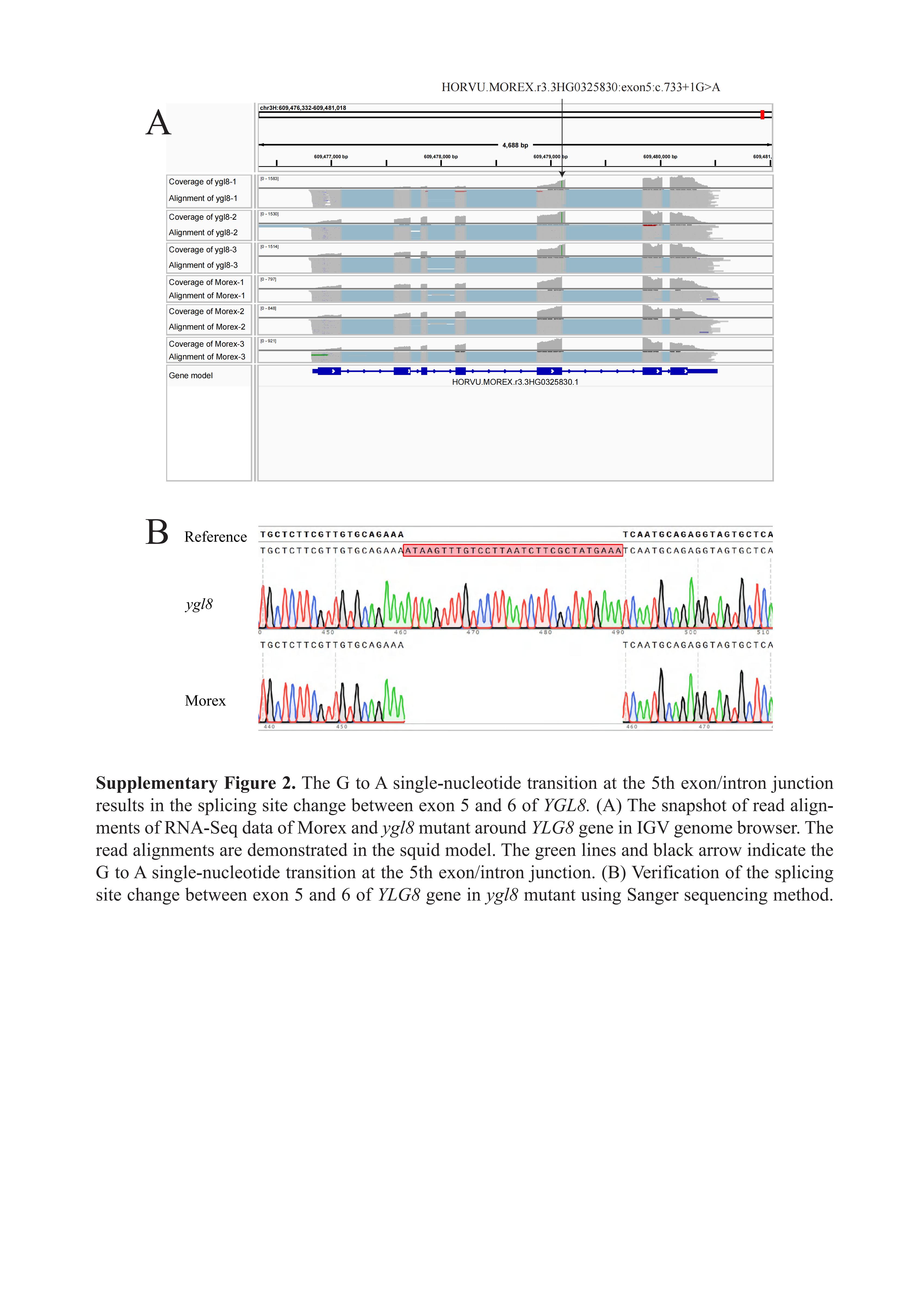

Supplement: Supplementary file 3 [file Image_2.jpeg]

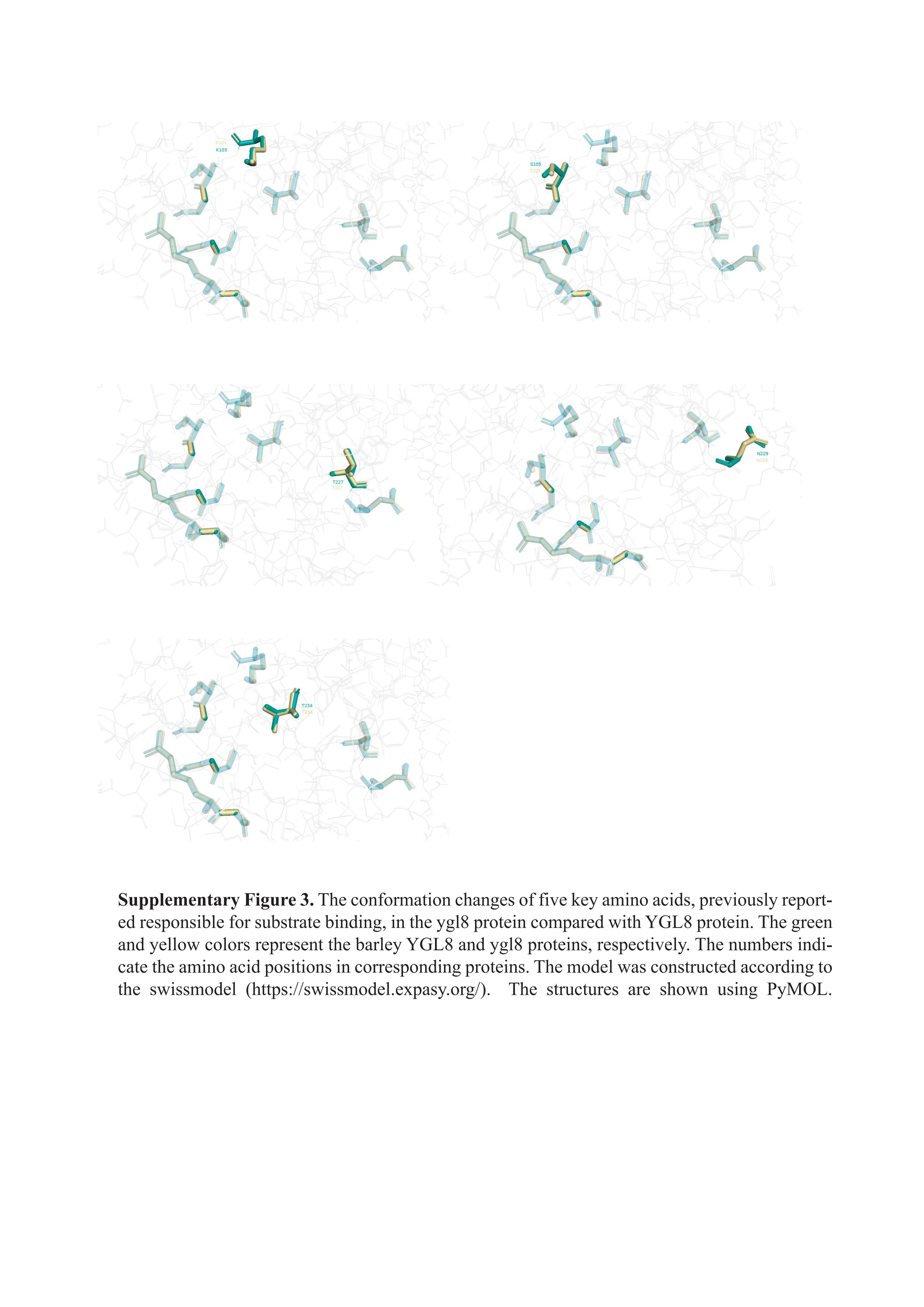

Supplement: Supplementary file 4 [file Image_3.jpeg]

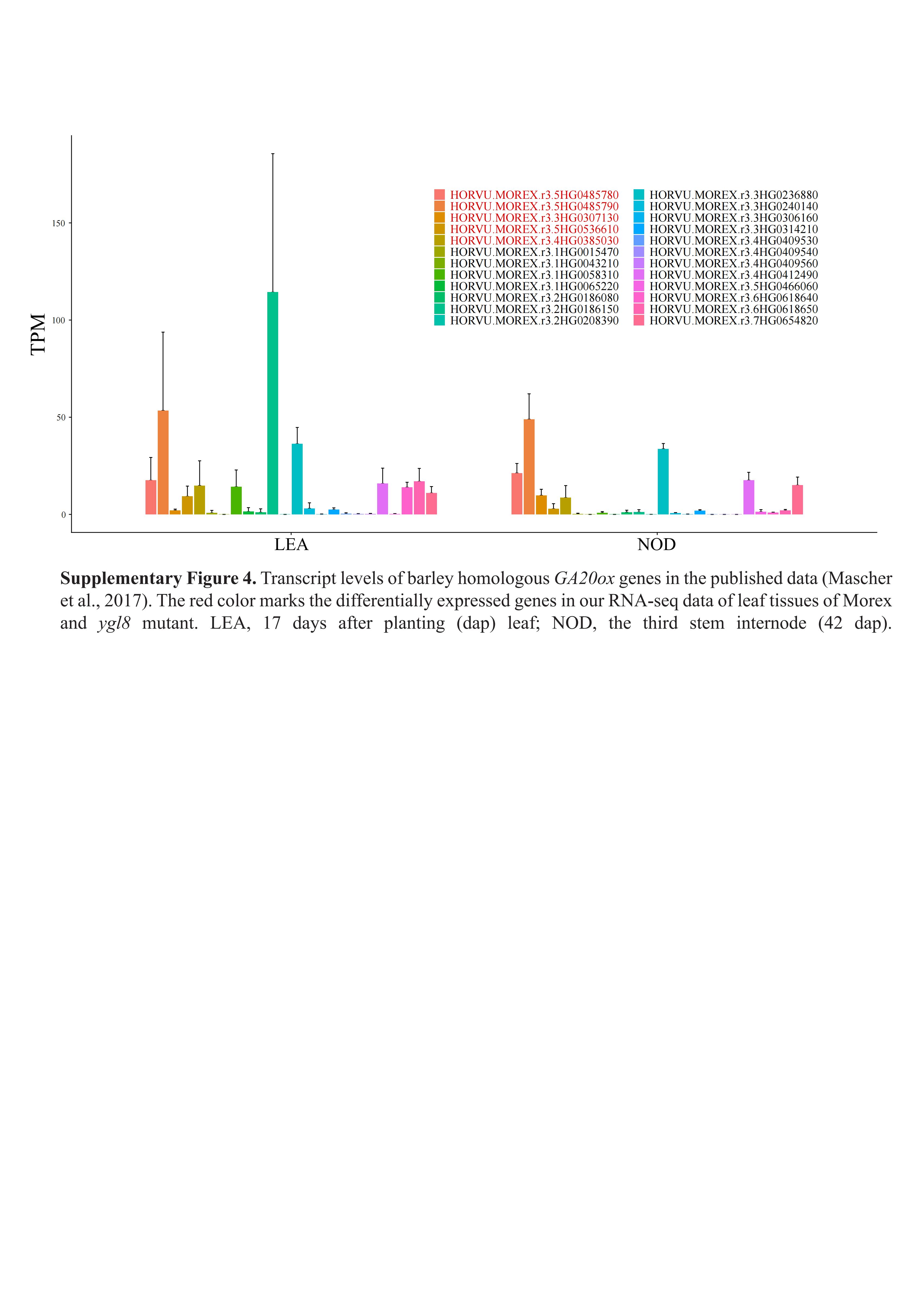

Supplement: Supplementary file 5 [file Image_4.jpeg]

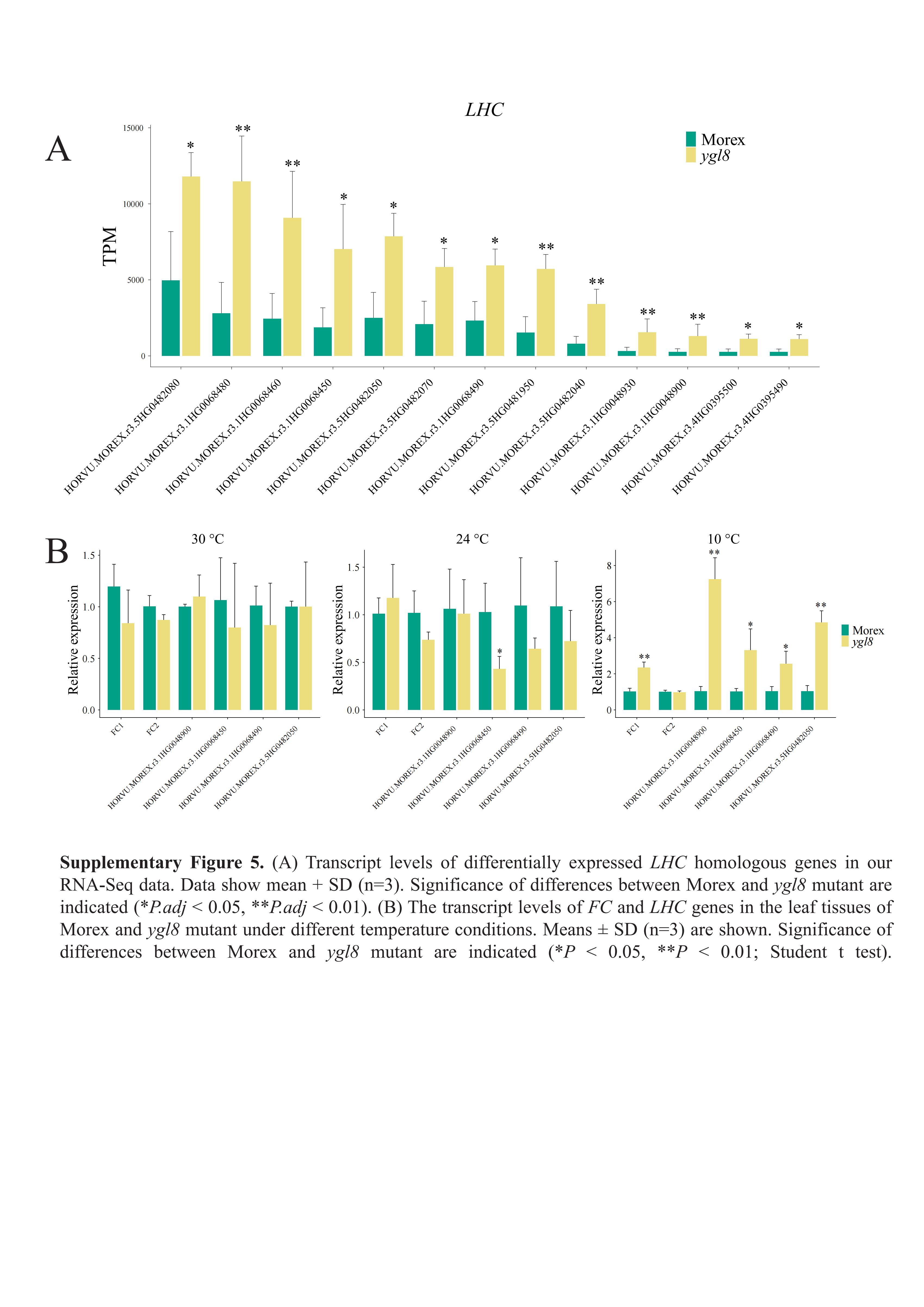

Supplement: Supplementary file 6 [file Image_5.jpeg]
